# Designing Self-Sustainable Icephobic Layer by Introducing a Lubricating Un-Freezable Water Hydrogel from Sodium Polyacrylate on the Polyolefin Surface

**DOI:** 10.3390/polym13071126

**Published:** 2021-04-02

**Authors:** Junqi Shi, Chongjian Cao, Lu Zhang, Yiwu Quan, Qingjun Wang, Hongfeng Xie

**Affiliations:** 1 Key Laboratory of High Performance Polymer Materials & Technology of Ministry of Education, School of Chemistry and Chemical Engineering, Nanjing University, Nanjing 210023, China; Lelouch_Shi@163.com (J.S.); adocaiel0923@163.com (C.C.); quanyiwu@nju.edu.cn (Y.Q.); 2 Systems Engineering Research Institute, Beijing 100094, China; lanjinglingsu@sina.com

**Keywords:** lubricant hydrogel, icephobic, polyacrylate, ice adhesion, self-sustainable

## Abstract

A convenient, environment-friendly, and cost-effective method to keep anti-icing for a long time was highly desirable. Slippery lubricant layers were regarded to be effective and promising for anti-icing on different surfaces, but the drought-out of lubricants and the possible detriments to the environment were inevitable. By combining super-high molecular weight sodium polyacrylate (H-PAAS) with polyolefin through a one-pot method, a self-sustainable lubricating layer with extremely low ice adhesion of un-freezable water hydrogel was achieved at subzero conditions. The lubricant hydrogel layer could auto-spread and cover the surface of polyolefin after encountering supercooled water, frost, or ice. Due to the reduction of storage modulus in the interface, the ice adhesion of the specimen surfaces was far below 20 kPa, varying from 5.13 kPa to 18.95 kPa. Furthermore, the surfaces could preserve the fairly low adhesion after icing/de-icing cycles for over 15 times and thus exhibited sustainable durability. More importantly, this method could be introducing to various polymers and is of great promise for practical applications.

## 1. Introduction

Due to precipitation and cold conditions, ice froze and accumulated on various horizontal and vertical surfaces, such as power lines [1], airplanes, heat exchangers [2], wind turbines [3], telecommunication networks [4], ships [5], cars, trains, and refrigerators [6]. Unnecessary icing caused not only an economic loss but also loss of life. Generally, the main methods of preventing or removing icing were classified into the active and passive methods [7]. Active methods were currently widely adopted, including installation of thermal system mechanical scraping [8] and spraying chemical deicers. However, many of these methods were expensive, high energy-consuming, and harmful to the environment. To avoid the shortcomings of active de-icing methods, passive methods, mainly including preventing the ice from adhering or promoting the ice sliding away under the wind, vibration, or solar radiation, had attracted the attention of scientists.

The traditional passive anti-icing surfaces mainly included nanostructured superhydrophobic surfaces (SHS) [9,10,11] and slippery liquid-infused porous surfaces (SLIPS). It had been proved that those surfaces had anti-icing properties since surface-free energy reduced [11]. According to the Cassie-Baxter model, the air trapped in the nano-texture beneath the droplet contributed to the low ice adhesion of SHS, extremely large water contact angle, and the icing delay [10,12]. However, moisture condensed in nanostructures and finally anchored the ice in high humidity and sub-zero condition, leading to the result that the ice adhesion strength increased. More seriously, the ice accumulated on the surface easily after the de-icing process destroying the textures [13,14]. Combined with thermal methods, the superhydrophobic/electrothermal method was another effective way to prohibit ice formation [15]. Then, the SLIPS coated with a functional liquid layer emerged as the times required to address the problem. Due to their hydrophobicity, low evaporation, and ice-strength, silicon and fluorinated liquids were widely studied as icephobic lubricant coatings [16,17,18,19]. Wang et al. [20] achieved a multi-fluorination strategy on polydimethylsiloxane (PDMS) matrix to enhance the liquid impalement resistance without sacrificing mechanical robustness. The ice-adhesion strength on all obtained surfaces could descend below 100 kPa, a critical value of an icephobic surface [18]. Surfaces with ultra-low ice adhesion strength below 20 kPa were a challenge. Because under this situation, ice adhered to material surfaces could be left off under natural forces, such as gravity, wind, or vibration without any extra energy consumed. Aiming at this goal, Golovin et al. [21] demonstrated that the ice layer could fall off completely by its gravity on the LIT PDMS (silicone B + 40 wt% silicone oil). Recently, much lower ice-adhesion strength, such as 2 kPa [22] and 0.4 kPa [21], was achieved. Although organ-oil infused slippery surface exhibited excellent anti-icing performance, the durability of oil was limited by how long the lubricant could be kept in the texture or block without leaking or evaporating [23]. Besides, the fluorinated liquid was high-cost and not environment-friendly [20]. Different from normal snow melting agents, strategies with novel materials, such as saline water, ethylene glycol, formamide, and water−glycerine, as an aqueous lubricating layer for anti-icing turned into view again. A new type of aqueous lubricating layer made from magnetic liquid [24], ferromagnetic liquid, antifreeze proteins [25], dopamine-modified hyaluronic acid [26], polyelectrolytes [27], polypeptide or hygroscopic polymers came into reality and showed excellent icephobic performance of ultra-low ice-adhesion strength as well as sustainable, self-healing, durable and environment-friendly advantages. Due to its water swelling, lower freezing point, fluidity, and no pollution, the hydrogel was potential for producing the water lubricating layer for shedding ice. In this paper, we designed a novel icephobic engineering material by blending polypropylene (PP) with super-high molecular weight sodium polyacrylate (H-PAAS) through a one-pot method. Only by one molding process, objects of various shapes with icephobic property were produced to form the lubricant hydrogel on the surface when contacting super cool water, frost, or ice in a cold environment. Both static and dynamic contact angles tests were performed on the specimen surface to investigate the formation of the lubricant hydrogel layer. The storage modulus (G’) of the hydrogel was tested through dynamic mechanical analysis (DMA). Besides, ice adhesion strength and anti-icing durability were gauged in a temperature-controllable chamber at −10 °C.

## 2. Materials and Methods

### 2.1. Materials

Polypropylene (PP, [CH_2_CH(CH_3_)]_n_) granules were obtained from Macklin Biochemical Co., Ltd. (Shanghai, China). Super-high molecular weight sodium polyacrylate powder (H-PAAS, [CH_2_CH(COONa)]_n_) with M_n_ of 30,000,000 was purchased from Heowns Biochemical Company (Tianjin, China).

### 2.2. Specimens Preparation

First, PP and H-PAAS were mixed for 5 min in a high-speed mixer (TPG 5IK60GN-CF, Induction Motors, Jiangsu Jinchengbang Precision Motor CO., Ltd., Suzhou, China), and then, the mixture was poured into a double screw extruder (CY-063, Sirocco Fan Industrial Co., Ltd., Guangzhou, China) to make PP-H-PAAS granules after mixing for 30 min at 180 °C. Finally, circular specimens (PP-H-PAAS) with a diameter of 7 cm and a thickness of 3 ± 0.2 mm were produced by an injection molding machine (YS-255, Aolai Trading Co., Ltd., Shenzhen, China) under a pressure of 0.7 MPa for 30 s. The H-PAAS mass fractions in the specimens were 0%, 5%, 10%, 15%, 20%, which were labeled as 0, 1, 2, 3, and 4, respectively.

### 2.3. Static and Dynamic Contact Angles and Delayed Icing Time

**Figure 1:**
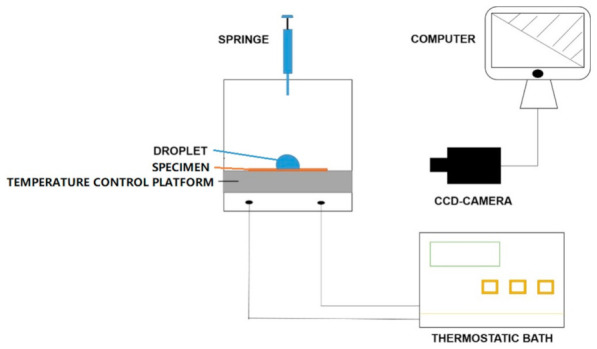
The schematic presentation of modified contact angle testing system.

As was shown in Figure 1, a specially modified optical angle meter (CAM 200, KSV Instrument Ltd., Helsinki, Finland) was used to gauge the contact angle (CA) in a temperature-controllable chamber [28,29]. Because the CA on such a hydrophilic surface would decrease in the time until the swelling had reached equilibrium. So, CA measurements were divided into two parts: static CA (SCA) and dynamic CA (DCA) measurements. The traditional method was used for measuring SCA within 1 min, and the photo intervals were set as 50 ms. However, the DCA within 2 h were gained by setting the photo intervals of 1 min during the CA achieved equilibrium, as shown in Figure S1 and Video S1. The structure details of the measurement chamber were shown in Figure S2. Because the whole CA measurement lasted for about 2 h, both the environment humidity and droplet evaporation would affect the water absorption. To avoid these influences, a rectangular-ambulatory water sink was used to maintain the relative humidity balance in the measurement chamber. So, when the measurement chamber was sealed during the CA test, the inner relative humidity of the chamber was near 100% [30], in other words, the air in the inner space of the chamber was saturated.

The air-conditioning system was composed of a thermostatic bath, temperature control platform, and a T-type micro temperature probe. The surface temperature of the specimen was accurately controlled from 25 to −15 °C.

According to our previous research [28], in situ observation of 8 μL water on a surface at −10 °C and delayed icing time (T_di_) were determined when transparent hemispherical shape changing to a dark opaque triangular-like peak.

### 2.4. Contact Angle Hysteresis (CAH)

The CAH also measured the specially modified optical angle meter as shown in Figure 1. Before the testing, the temperature control platform was replaced with another tiltable platform and the specimens were stuck on the tiltable platform one by one. Then, a water droplet of 24 uL was added to the specimen. During the characterization, the platform was tilted until the water droplet began to slide away. Finally, the advancing contact angle ($\theta_{Adv}$) and receding contact angle ($\theta_{Rec}$) were gained by the computer software. The $\theta_{Adv}$ minus $\theta_{Rec}$ was the CAH value.

### 2.5. Ice Adhesion Strength and Anti-Icing Durability

**Figure 2:**
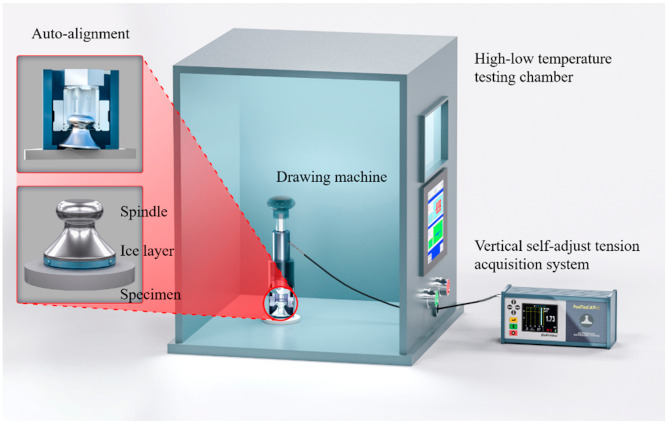
The schematic presentation of modified system for testing ice adhesion tension strength.

As shown in Figure 2, the iced specimens were in a temperature-controllable chamber (FH-408R, TuoDe Industrial Co., Ltd., Shanghai, China) with a vertical self-adjust tension acquisition system (PosiTest AT-A, DeFelsko Corporation, New York, NY, USA) according to ASTM D4541. The adhesion strength could be acquired through the following formula:

(1) $$\tau_{ice}=\frac{F}{A}$$

where *A* was the ice covering area, *F* was the vertical tension. Given that $\tau_{ice}$ was dependent on the thickness of the ice layer [31], a plastic ring with a diameter of 50 mm was stuck on the specimen in advance, and the ice thickness was precisely controlled at 2.0 mm by inserting three globes of 2.0 mm diameter apart to form a small gap between the spindle and the specimen. Then, a medical injector was used to inject enough deionized water into the gap and the spindle-water-specimen “sandwich” was deposited in the temperature-controllable chamber with the temperature of −10 °C and the relative humidity of 0% for icing. The deionized water was sealed between the spindle and specimen without being influenced by the relative humidity. For testing the low ice adhesion strength, spindles with 50 mm diameter were selected. All the ice adhesion experiments were performed at −10 °C in the temperature-controllable chamber. The icing time for both ice adhesion and icing/de-icing cycle tests was 20 h, which was believed to be ample for complete icing. When testing ice adhesion, a drawing machine was used to pull the spindle with an ice layer beneath itself off the specimen surface. The open bottom part of the drawing machine would be able to get a good grip on the top hump part of the spindle. Then, the motor drove the open bottom part and the spindle to raise at 2 kPa/s or 20 kPa/s until the spindle, together with ice, was separated from the specimen surface. The drawing machine had the function of auto-alignment to ensure throughout verticality. The dial would show the maximum value of vertical extension force that represented the ice adhesion strength.

The icing/de-icing cycle test was carried out on the Specimen 3 by repeating the same procedures of ice adhesion measurement for 15 times. Each time, after the drawing machine was used to divorce the spindle and the ice layer from the center of the specimen surface, another three globes were inserted to control the gap height at 2 mm. Afterward, the deionized water was injected to form an ice layer again for next icing/de-icing cycle. After completing the de-icing process, the ice layer still adhered to the bottom of the spindle as shown in Figure S3.

### 2.6. Surface Morphology and Elemental Analysis

The surface morphology and elemental analysis of the specimens were recorded by the scanning electron microscope (SEM, S-4800, Hitachi, Japan) equipped with an energy diffraction spectrum (EDS) at 5.0 kV. Besides, the roughness of the specimen surfaces was tested by the atomic force microscope (AFM, Dimension Edge, Bruker, Germany). The scan size was 20 µm × 20 µm, the scan rate was 60 µm/s and sample lines were 256. The chemical composition was further investigated by X-ray photoelectron spectroscopy (XPS, K-Alpha, Thermo Scientific Co., Ltd., Waltham, MA, USA). The number of scans was 5, the source gun type was Al K Alpha, the spot size was 400 µm, and the energy step size was 0.100 eV.

### 2.7. Water Swelling Ratio

The water swelling ratio (*S*) was defined as

(2) $$S=\frac{w_{2}-w_{1}}{w_{1}}$$

where $w_{1}$ and $w_{2}$ respectively referred to the weights of the H-PAAS powder and the hydrogel after absorbing sufficient water [32]. After immersing in the water for 4 h, the hydrogel on the PP-H-PAAS surface was collected and the value of $w_{1}\;$ was obtained. $w_{2}$ was obtained from drying the collected hydrogel in a hot oven at 120 °C for 2 h.

### 2.8. DMA

**Figure 3:**
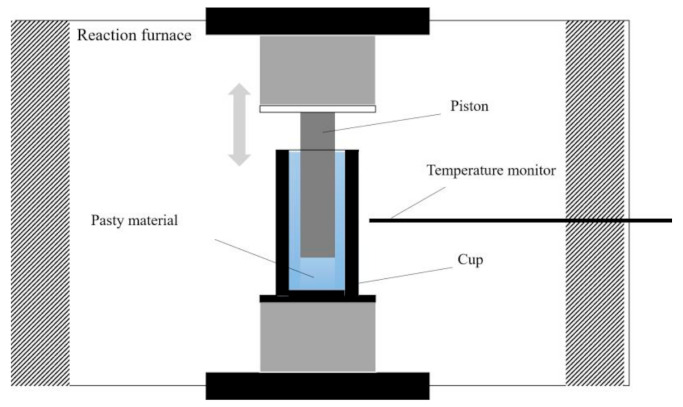
The schematic presentation of the dynamic mechanical analyzer.

As shown in Figure 3, by pouring the collected hydrogel into the cup of the dynamic mechanical analyzer (DMA+ 450, 01DB-MetraviB, Lyon, France), the pasty material model was selected to testing the G’ of H-PAAS hydrogel or water, respectively, when temperature varied from −30 °C to 25 °C at 0.1 °C/min with a frequency of 1 Hz. The piston diameter was 7 mm, the internal cup diameter was 10 mm and the sheared height was 20 mm.

## 3. Results and Discussion

### 3.1. Surface Morphology

**Figure 4:**
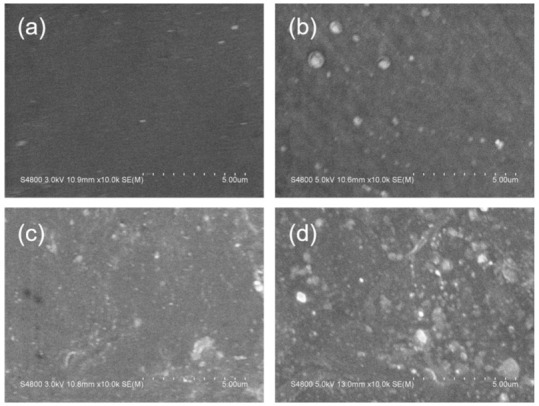
SEM images of PP-H-PAAS surfaces; (**a**–**d**) were the smooth surfaces of specimen 1, 2, 3, 4, respectively.

Figure 4 showed SEM images of PP-H-PAAS surfaces with no rough structures as compared to the typical superhydrophobic surfaces [32]. The PP-H-PAAS surfaces were both flat and hydrophilic. It was suitable for the aqueous slippery layer to extend and flow on such surfaces in Wenzel’s model without any barrier.

**Figure 5:**
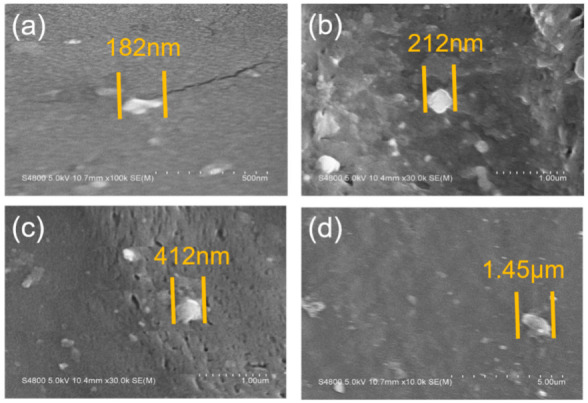
The size of H-PAAS islands scattered in the PP sea; (**a**–**d**) represented four kinds of H-PAAS island sizes.

The situation of the H-PAAS phase dispersed in the continuous PP phase was suitable for the typical sea-island model. Figure 5 illustrated the size of H-PAAS islands scattered in the PP sea in SEM images. The H-PAAS powder aggregated together to form bright white particles (islands), which dispersed in the PP phase (sea). The particle size of H-PAAS fluctuated from several hundred nanometers to several microns, whose average value was approximately 500 nm. Note that the particle size of H-PAAS was suitable for forming a continuous aqueous slippery layer when absorbing super cooler water or melting frost and ice in a cold environment [33].

**Figure 6:**
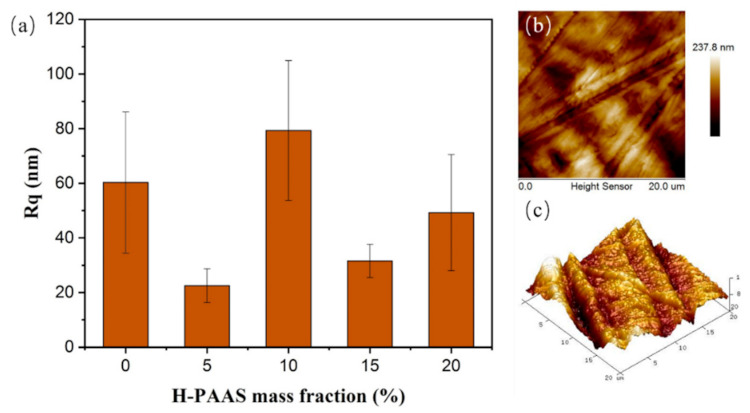
The Rq of specimens with different H-PAAS mass fractions (**a**). 2D image (**b**) and 3D image (**c**) of Specimen 3.

The surface roughness of the specimens tested by AFM was shown in Figure 6. The root mean square roughness (Rq) varied from 22.5 nm to 79.3 nm, which was independent of the H-PAAS mass fraction. It was worthy to note that the injection molding machine was used to inject the melting PP-H-PAAS into the circular plate under a pressure of 0.7 MPa during specimen fabrication. This pressure was high enough to cause inevitable mechanical scratches during the injection process as shown in Figure 6b,c. Besides, these scratches appeared randomly on the specimen surfaces. Therefore, there was no linear relationship between Rq and H-PAAS mass fraction.

### 3.2. Static and Dynamic Contact Angles

**Figure 7:**
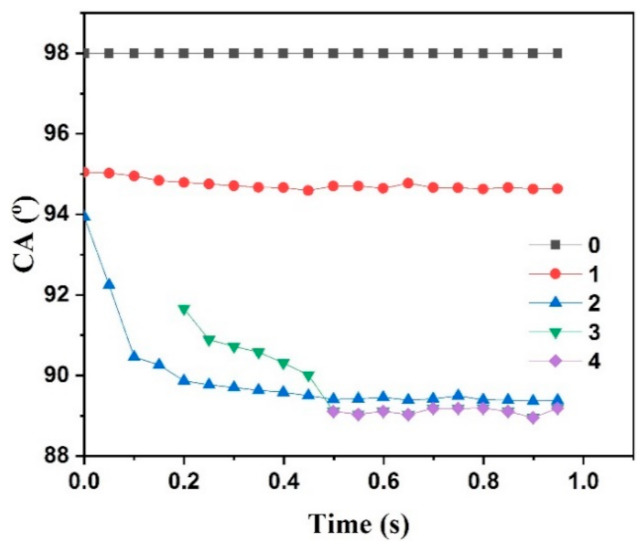
Static contact angles as a function of time for specimens with different H-PAAS mass fractions.

Figure 7 showed the CA as a function of time for specimens. To investigate the instantaneous CA as soon as the water droplet contacted the specimen surface, the photo intervals of the high-speed camera were set as 50 ms. So, the first computable CA could be determined by checking these photos recorded in 1 s. Then, the first CA value that was the starting point of every curve, as shown in Figure 7, could also be accurately calculated. That was the reason why every curve did not start at 0.0 s. These CA values of the starting points were the traditional SCA. It was more difficult for the water droplet to form a stable hemispherical shape as quickly as possible when the H-PAAS mass fraction increased. So, the time of starting point in the curves was delayed for PP-H-PAAS with a higher H-PAAS mass fraction. The curves of the PP-H-PAAS surface with lower H-PAAS mass fractions decreased at first and gradually leveled out, while the curve of the pure PP surface kept unchangeable.

As shown in Table 1, static contact angles of specimens at room temperature decreased from 98.0° to 89.1° as the H-PAAS mass fraction increased from 0% to 20%, which indicated that the origin hydrophobicity of PP was changed to hydrophilicity due to the addition of H-PAAS. With the existence of H-PAAS, the surface could easily absorb water to extend and form a flowing hydraulic gel layer. The SCAs of four compound specimens were close to the CA value calculated from Cassie-Baxter’s equation (Equation (3)), while the SCAs of PP and PAAS were 98.0° and 0.0°, respectively. Being made from two parts of hydrophobic PP and hydrophilic H-PAAS, the compound surface was just like H-PAAS islands dispersed in the PP sea as shown in Figure 5. Therefore, the wetting model that followed Cassie-Baxter’s equation was similar to a superhydrophobic surface. The only difference was that SHS contained hydrophobic air clumbers while the PP+H-PAAS surface had hydrophilic H-PAAS dots.

(3) $$\cos\theta_{\mathrm{PP}-H-\mathrm{PAAS}}=f_{\mathrm{PP}}\times \cos\theta_{PP}+f_{H-\mathrm{PAAS}}\times \cos\theta_{H-\mathrm{PAAS}}$$

where $f_{\mathrm{pp}}$ and $f_{H-\mathrm{PAAS}}$ were the mass fraction of PP and H-PAAS, respectively.

**Figure 8:**
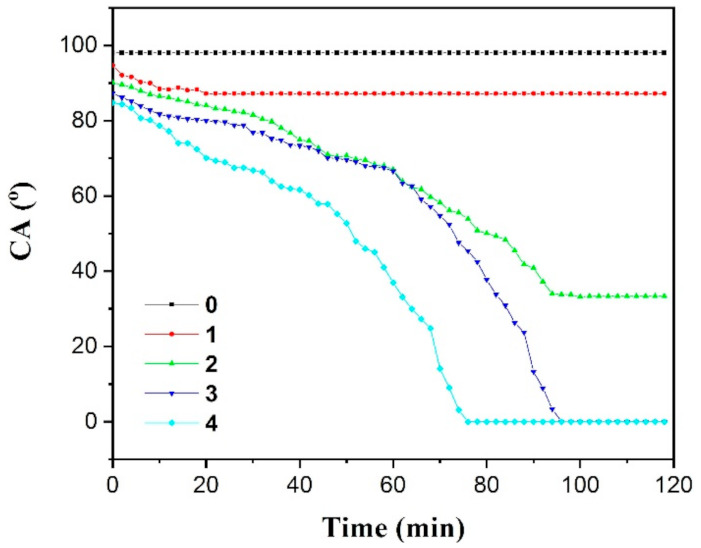
Dynamic contact angle versus time curves of PP and its H-PAAS compounds.

As shown in Figure 8, the DCAs of pure PP kept unchangeable at room temperature during all time. However, for compound specimens, the effect time on the DCA was dependent on the H-PAAS concentration. The DCA of the compound with 5% H-PAAS decreased slowly in time and leveled out after 20 min. With the further increase of H-PAAS concentration, the DCA decreased rapidly in both time and H-PAAS concentration, while the time for DCA to level out decreased in the H-PAAS concentration. At first, the isolated H-PAAS absorbed water and auto-spread. Consequently, the hydrophilic area gradually connected as time went by. Not surprisingly, therefore, the DCA of compound specimens decreased in both time and H-PAAS concentration. Due to its super-high molecular weight with many long main chains and side chains, it takes a really long time for H-PAAS to absorb water until main chains and side chains unfolded completely [34].

**Figure 9:**
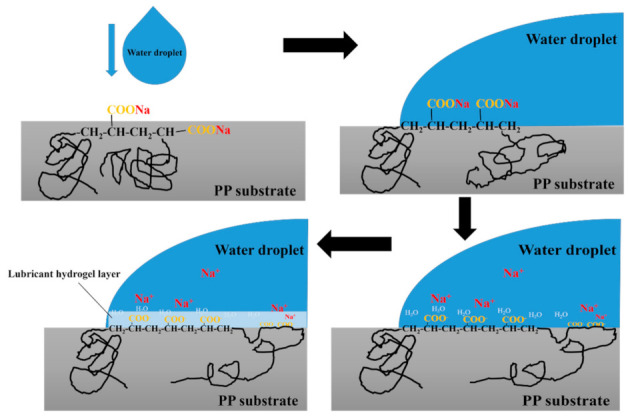
Scheme for the formation of lubricant hydrogel layer on the interface between water and specimen.

Note that, during the absorption, there was an H-PAAS lubricant hydrogel layer (H-PAAS-LHL) formed in the interface of specimen and water. Figure 9 illustrated a scheme for the formation of a lubricant hydrogel layer on the interface between water and specimen. Before adding water, the long H-PAAS main chains were embedded in the PP substrate field and only a small part of the whole polymer was exposed to the specimen surface, which contained some hydrophilic salts (-COONa) at the end of the side chains. After adding water to the specimen surface, the water droplet not only contacted the PP chains, but also the side chains of H-PAAS. Then, the hydrophilic salts (-COONa) began to ionize and release Na^+^. At the same time, the remaining -COO^−^ began to capture and combine with water molecules. Those captured water molecules were fixed on the top surface of the specimen, in other words, the interface between the water phase and the specimen phase. With the number of fixed water molecules increasing, a continuous hydrogel layer to separate water and specimen formed.

**Figure 10:**
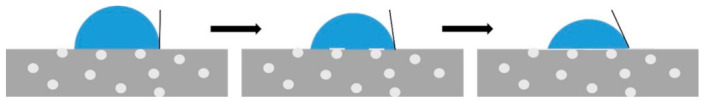
Scheme for auto-spread of H-PAAS-LHL between water and surface.

The emergence of the lubricant hydrogel layer lowered the DCA, so it took more than one hour for the whole CA equilibrium process, as shown in Figure 8. At last, the spontaneously spreading H-PAAS-LHL expanded on the specimen surface to form a hydrogel film of the H-PAAS layer, during which the DCA of each surface also kept decreasing. Until the H-PAAS on the interface absorbed enough water to form a relatively saturated station, the area of the hydrogel stopped increasing at the maximum value, representing that the hydrogel had completely auto-spread. That is why a plateau appeared at the end of every curve in Figure 8. With the mass fraction of H-PAAS increasing, the initial DCA value decreased. It was seen that by adding 5% H-PAAS, the final CA only declined to about 88° as compared to 98° of the pure PP. However, when the mass fraction of H-PAAS increased to 10%, the final DCA lowers to about 33.3°, which was 64.7° lower than that of the pure PP. As discussed previously, DCA declined faster in time as the H-PAAS mass fraction increased. When the H-PAAS mass fraction reached 15% and 20%, it took 96 min and 77 min, respectively, for the DCA to level out. As shown in Figure 10, when the compound surface met water, the top surface of single H-PAAS dots absorbed water, and then, extended along the surface. Finally, nearly all H-PASS dots connected each other by forming a flowing hydraulic gel layer. The spread speed of the hydraulic gel layer was controlled by the distance of H-PAAS dots and the water absorption. So, the compound materials of 20% H-PAAS spent the minimum time to cover the surface by a hydraulic gel layer. Moreover, the continuous decrease in the DCA was helpful to extend the freezing time, because the contact line kept decreasing during the whole auto-spreading process and inhibited small ice crystals joining into blocks, which prevented the surface from icing.

### 3.3. Dynamic Mechanic Analysis of Water and Hydrogel

**Figure 11:**
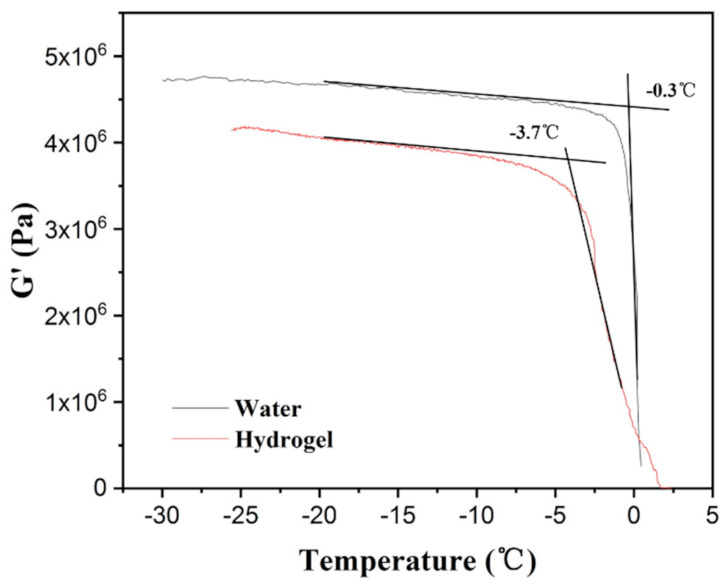
G’ versus temperature curves of water and hydrogel.

According to Equation (1), the water swelling ratio was about 300. To determine the dynamic mechanical properties of the hydrogel, the hydrogel on the surface of specimens was collected in the cup of the DMA instrument. As shown in Figure 11, the G’ value of hydrogel was lower than that of water and the difference remained at about 0.6 MPa when the temperature was below 0 °C. The freezing points of water and hydrogel were −0.3 °C and −3.7 °C, respectively, indicating that the freezing point of water lowered when the water formed an unfrozen hydrogel layer due to the existence of H-PAAS. The hydrogel reduced the G’ of the interface between ice and specimen. The lower G’ value represented lower stiffness and higher flexibility of a material, which led to less resistance to deformation including elongation and shear. This was the reason why the hydrogel layer decreased greatly the ice adhesion strength. Golovin et al. [35] demonstrated that low interfacial toughness within the ice allowed ice to be shed by self-weight.

### 3.4. Anti-Icing Property

**Figure 12:**
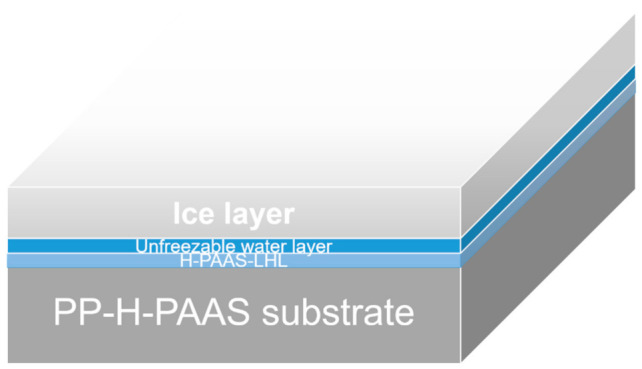
Scheme of H-PAAS lubricant hydrogel layer (H-PAAS-LHL).

Table 2 summarized the icing time and T_di_ of specimens. The T_di_ at −10 °C increased in the H-PAAS mass fraction. For the SHS, the higher and bigger the contact angle was, the longer the delay time of ice formation [36]. Quite different from that, generally, smooth and hydrophilic surface iced easily and the delay icing time was short. Indeed, the PP-H-PAAS specimen was an anion-rich polymeric surface. When the specimen surface met super cooler water on its surface, the -COO^−^ of H-PAAS exposed to the external surface quickly to capture water under the side-chain extension motion and extended an extra thin lubricant hydrogel layer between the water phase and substrate material phase as seen in Figure 12. During this extension, sodium ion of H-PAAS diffused into the lubricant hydrogel layer. The more Na^+^ of polyelectrolyte proliferated in the lubricant hydrogel layer, the lower the freezing point due to the hydration [37,38,39]. Notably, the lubricant hydrogel layer suppressed the heteronuclear crystallization, resulting in a much lower crystallization temperature and a longer icing time during the cooling process. The hydrogel layer that showed a small DCA value (less than 90°) was definitely a high surface energy layer. For a general high surface energy layer, it would lower the critical Gibbs free energy of crystallization. However, as a key icephobic material, H-PAAS generated a special hydrogel layer. On the one hand, with the addition of H-PAAS, there were numerous Na^+^ and -COO^−^ having entered the hydrogel layer and these ions could reduce the freezing point to delay icing. On the other hand, the main chains of H-PAAS were long enough to take nearly 2 h for these main chains to stretch completely when encountering water, which led to the continuous decrease in the DCA value as shown in Figure 8. The hydrogel layer was not completely formed in one second but in 2 h until the main chains absorbed enough water. Further, during this absorption process, Na^+^ and -COO^−^ of side chains of H-PAAS kept entering the hydrogel layer. The decrease in the DCA value also meant the decrease of the contact line, which resulted in the consequence that the interface area between the water phase and specimen phase kept increasing. This could damage the formed ice crystallization and delayed icing. Therefore, the super cooler water near the lubricant hydrogel layer was difficult to freeze and the icing time was delayed. Therefore, Specimen 4 attained a super long unfrozen level of 3048 s, which was 126-fold of PP.

**Figure 13:**
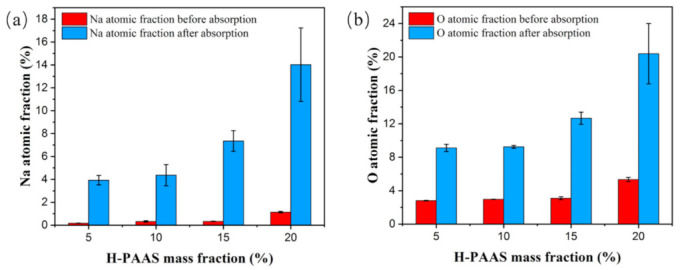
Na (**a**) and O (**b**) atomic fractions before and after water absorption.

**Figure 14:**
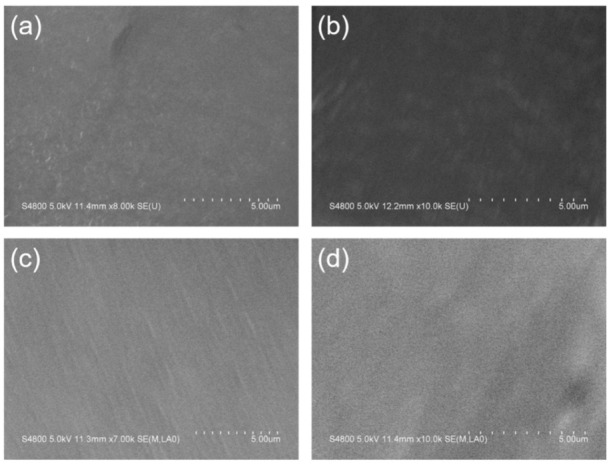
H-PAAS-LHL covered the whole PP-H-PAAS surfaces. Noted that the image (**a**–**d**) referred to sample 1 to 4 respectively, as compared to the surfaces in Figure 2 (**a**–**d**).

As shown in Figure 13, the elemental distributions of Na and O increased rapidly after absorbing water on the four PP-H-PAAS surfaces through the assistance of EDS, the maps of EDS could be seen in Figures S4–S7. This indicated that the H-PAAS chains transferred from their original position to the whole compound surface after absorbing enough water. When the H-PAAS chains, more specifically, the side chains of H-PAAS, encountered with water, the Na^+^ and -COO^−^ combined with water molecules began to form an H-PAAS-LHL and spread to further sites which drove the H-PAAS main chains to move, contributing to the auto-spread of H-PAAS-LHL. In consequence of this, both H-PAAS islands and PP sea were covered by a smooth H-PAAS-LHL as seen in Figure 14, while the original H-PAAS islands (Figure 4) were missing [40]. Further, the enrichment of Na^+^ iron on the topmost H-PAAS-LHL layer lowered the freezing point and formed an ultra-thin un-freezable water layer. The slippery layer beneath ice decreased the ice adhesive strength of the surface of compound materials.

**Figure 15:**
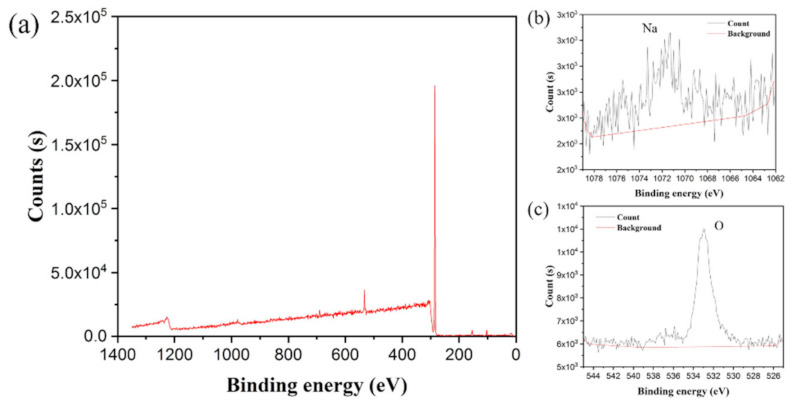
XPS spectrum of Specimen 3 before water absorption: the whole scanning region (**a**); the region of Na (**b**) and the region of O (**c**).

**Figure 16:**
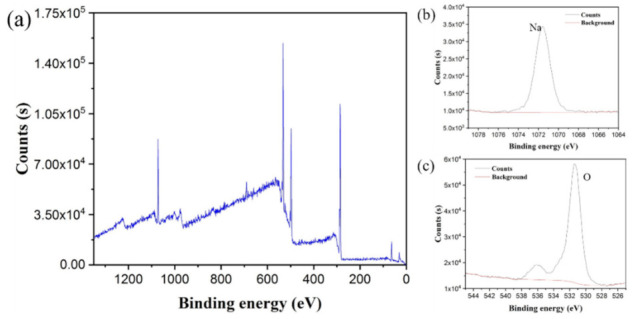
XPS spectrum of Specimen 3 after water absorption: the whole scanning region (**a**); the region of Na (**b**) and the region of O (**c**).

To intensively investigate the detailed chemical composition changes before and after water absorption, XPS tests were performed. Figure 15 and Figure 16 presented the XPS spectra of Specimen 3 before and after water absorption. The Na and O atomic fractions were calculated by the integral of the area between the peak curve and background curve, as shown in Table 3. After the water absorption, the Na atomic fraction increased to 6.11%, which was 29.1 times higher than the original value. Correspondingly, the O atomic fraction increased to 26.94%, which was 7.6 times higher than the original one. Because when the specimen surface encountered the water, the salt (-COONa) of the H-PAAS side chains released Na^+^ ions which was of less weight and could move randomly in the water phase at a higher speed. However, the -COO^−^ anions were fixed on the H-PAAS main chains by the C-C binding, so the displacement of -COO^−^ anions was limited. That is why the migration speed of Na was more rapid than that of O.

**Figure 17:**
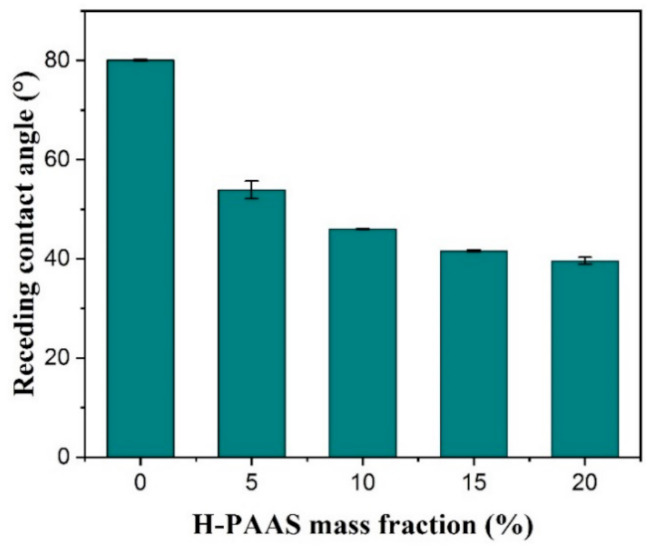
Receding contact angles of the surfaces of PP and PP-H-PAAS.

Receding contact angle was another important factor in close relation with the ice adhesion strength. Figure 17 showed the correlation between receding contact angle and H-PAAS mass fraction. Meuler et al. [41] studied the correlation between wettability and ice adhesion of 21 slippery coatings and found the negative relationship between $\theta_{Rec}$ and $\tau_{ice}$. However, our experiment presented a positive relationship. Because the PP-H-PAAS was not a kind of low surface energy material, the $\theta_{Rec}$ was also less than 60°, not like the SHS whose $\theta_{Rec}$ was supposed to exceed 150°. Nevertheless, there was still a decreasing trend in $\theta_{Rec}$ when the H-PAAS mass fraction increased due to the formation of an H-PAAS-LHL. Because the formation of an H-PAAS-LHL was the process of H-PAAS chains capturing water molecules, when there were more H-PAAS chains on the interface of the specimen with higher H-PAAS mass fraction, it was more difficult for the water droplet to spread. Since the H-PAAS had a very large value of water swelling ratio, a small amount of water droplet was more likely to be absorbed by the specimen surface to form an H-PAAS-LHL, rather than move freely, which contributed to an increase in the surface lubricity and icephobicity. So, the lower the $\theta_{Rec}$ was, the larger the H-PAAS-LHL area was. Consequently, the specimen surface became more lubricant and softer.

**Figure 18:**
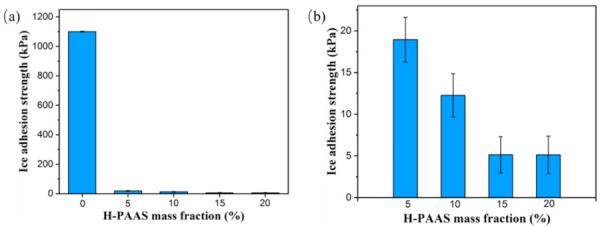
The ice adhesion strength of the surfaces of PP and PP-H-PAAS; (**a**) represented the ice adhesion of specimen 0 to 4, (**b**) represented the ice adhesion of specimen 1 to 4.

The ice adhesion strength of the specimens was shown in Figure 18. In comparison to the bare Al surface with a $\tau_{ice}$ of more than 1.56 MPa [42], the $\tau_{ice}$ on the PP surface was also fairly strong (over 1100 kPa). In this case, it was laborious to clean the icy PP surface in the low-temperature environment. Although the PP surface was merely mildly hydrophobic for its CA reached around 98°, the water droplets could hang on the surface because of its high adhesion strength. After mixing with 5% H-PAAS, however, the specimen surface exhibited an apparent anti-icing property. The $\tau_{ice}$ decreased to 18.95 kPa with a decline of 98.28%, and the surface became a great icephobic surface that could promise to complete the de-icing process under the force of a nature strong breeze. As shown in Figure 16b, the ice adhesion of four specimens was all below 20 kPa, which was a critical value of ice shed off under a natural force. This demonstrated that all the designed surfaces could be applied in snow weather with the self-deicing function. Interestingly, there came a plateau for $\tau_{ice}$ that emerged at the H-PAAS mass fraction of 15%. The adhesion strength maintained the value of 5.14 kPa even if H-PAAS mass content was increased to 20%. It could assume that the H-PAAS was saturated for the adhesion strength of the H-PAAS-LHL when the H-PAAS mass fraction reached 15%.

**Figure 19:**
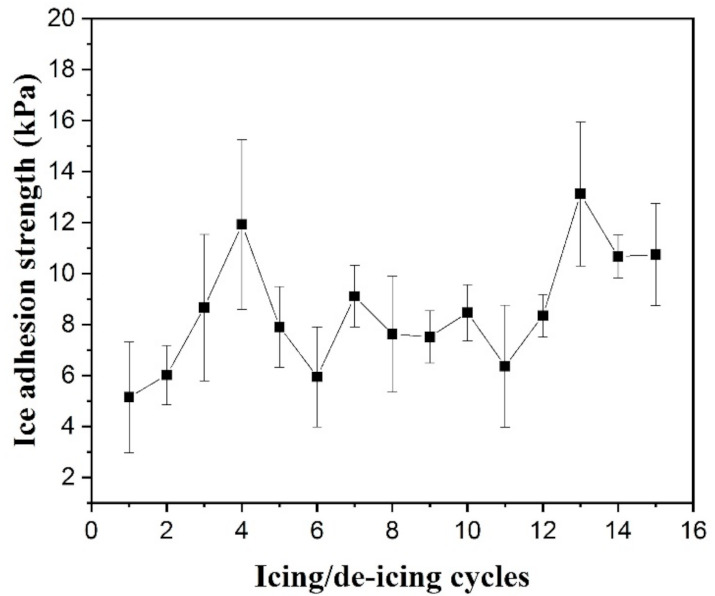
Ice adhesion change during icing/de-icing cycles.

Durability was another essential aspect considered for an icephobic material [43]. To evaluate the durability of PP-H-PAAS materials, icing/de-icing cycle experiments (15 times) were implemented on Specimen 3, which exhibited the minimal $\tau_{ice}$ value as discussed previously. After the durability test, the adhesion strength had slightly risen from 5.14 kPa to about 10 kPa as shown in Figure 19. As compared to the threshold for self-removal of snow and ice, the ice adhesion was still fairly low after cycles for 15 times, which was even lower than the half value. So, the H-PAAS-PP surface performed the outstanding durability.

## 4. Conclusions

A novel icephobic engineering raw material was introduced by blending polypropylene with super-high molecular weight sodium polyacrylate through a one-pot method. Objects with icephobic properties were produced by simple molding processing to form a lubricant hydrogel on the surfaces even in a cold environment. The designed surfaces performed greatly low ice adhesion properties. Compound materials with 20% H-PAAS attained a super long unfrozen level of 3048 s, which was 126 fold of the pure PP. The adhesion strength on the sample surfaces was all below 20 kPa. The minimal value (5.14 kPa) was obtained with 15% H-PAAS being added. Furthermore, the surfaces could maintain this low adhesion level during icing/de-icing cycles for over 15 times. Therefore, a convenient, environment-friendly, self-sustainable, and cost-effective method on different materials provided more opportunities for producing polymeric materials with durable and anti-icing properties.

## Figures and Tables

**Table 1:** Static contact angles of specimens.

**Specimen**	$CA_{\mathrm{pp}}$	$CA_{H-\mathrm{PAAS}}$	$f_{\mathrm{pp}}$	$f_{H-\mathrm{PAAS}}$	$CA_{\mathrm{PP}-H-\mathrm{PAAS}}$	$CA_{\mathrm{Tested}}$
0	98.0	0.0	1.00	0.00	98.0	98.0
1	98.0	0.0	0.95	0.05	95.8	95.0
2	98.0	0.0	0.90	0.10	93.5	93.9
3	98.0	0.0	0.85	0.15	91.3	91.7
4	98.0	0.0	0.80	0.20	89.0	89.1

**Table 2:** Icing time and delay icing time of specimens.

Specimen	Icing Time/s	Delayed Icing Time/s
0	24 ± 3.2	-
1	29.8 ± 6.4	5.8 ± 6.4
2	69.8 ± 9.1	45.8 ± 9.1
3	106.8 ± 5.9	82.8 ± 5.9
4	3072 ±376.4	3048 ± 376.4

**Table 3:** Na and O atomic fractions before and after water absorption.

Process Status	Na Atomic Fraction/%	O Atomic Fraction/%
Before water absorption	0.21	3.53
After water absorption	6.11	26.94

## Data Availability

Excluded.

## Supplementary Materials

The following are available online at https://www.mdpi.com/article/10.3390/polym13071126/s1. Figure S1: CAs of Specimen 3 at different time. Figure S2: Structure details of the temperature-controlled measurement chambers. Figure S3: Ice layers adhered to the spindles after de-icing. Figure S4 to S7: EDS spectra of the specimens with different H-PAAS mass fractions. Video S1: CA measurement of Specimen 3.
