# Supplementary material for: Designing Self-Sustainable Icephobic Layer by Introducing a Lubricating Un-Freezable Water Hydrogel from Sodium Polyacrylate on the Polyolefin Surface
Source: Polymers (Basel). 2021 Apr 2;13(7):1126. doi: 10.3390/polym13071126 (PMC8037279; doi:10.3390/polym13071126)
Supplement: Supplementary file 1 [file polymers-13-01126-s001.pdf]

Supplementary materials

Designing Self-Sustainable Icephobic Layer by Introducing a Lubricating Un-Freezable Water Hydrogel from Sodium Polyacrylate on the Polyolefin Surface

Junqi Shi [1], Chongjian Cao [1], Lu Zhang [2], Yiwu Quan [1], Qingjun Wang [1,*] and Hongfeng Xie [1,*]

[1] Key Laboratory of High Performance Polymer Materials & Technology of Ministry of Education, School of Chemistry and Chemical Engineering, Nanjing University, Nanjing 210023, China; Lelouch_Shi@163.com (J.S.); adocaiel0923@163.com (C.C.); quanyiwu@nju.edu.cn (Y.Q.)

[2] Systems Engineering Research Institute, Beijing 100094, China; lanjingling-su@sina.com

Corresponding authors:

E-mails: njuwqj@nju.edu.cn (Q. Wang) and hfxie@nju.edu.cn (H. Xie).

Tel.: (86)-25-89682568

Fax: (86)-25-89681907.

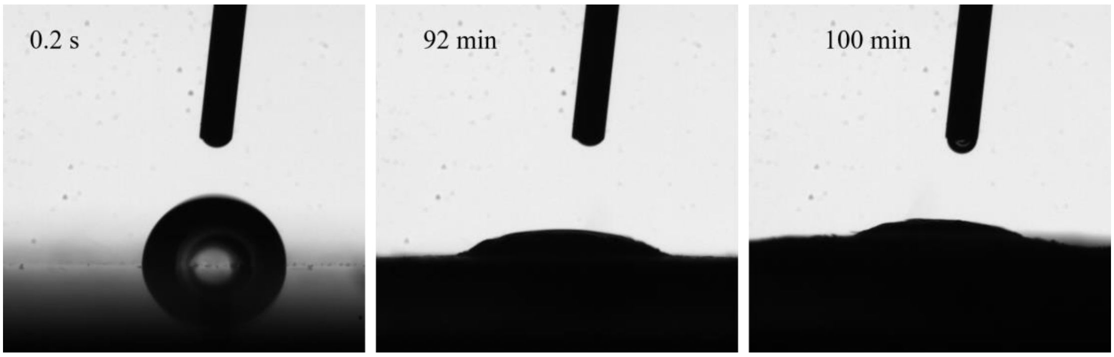

Figure S1. CAs of Specimen 3 at different time.

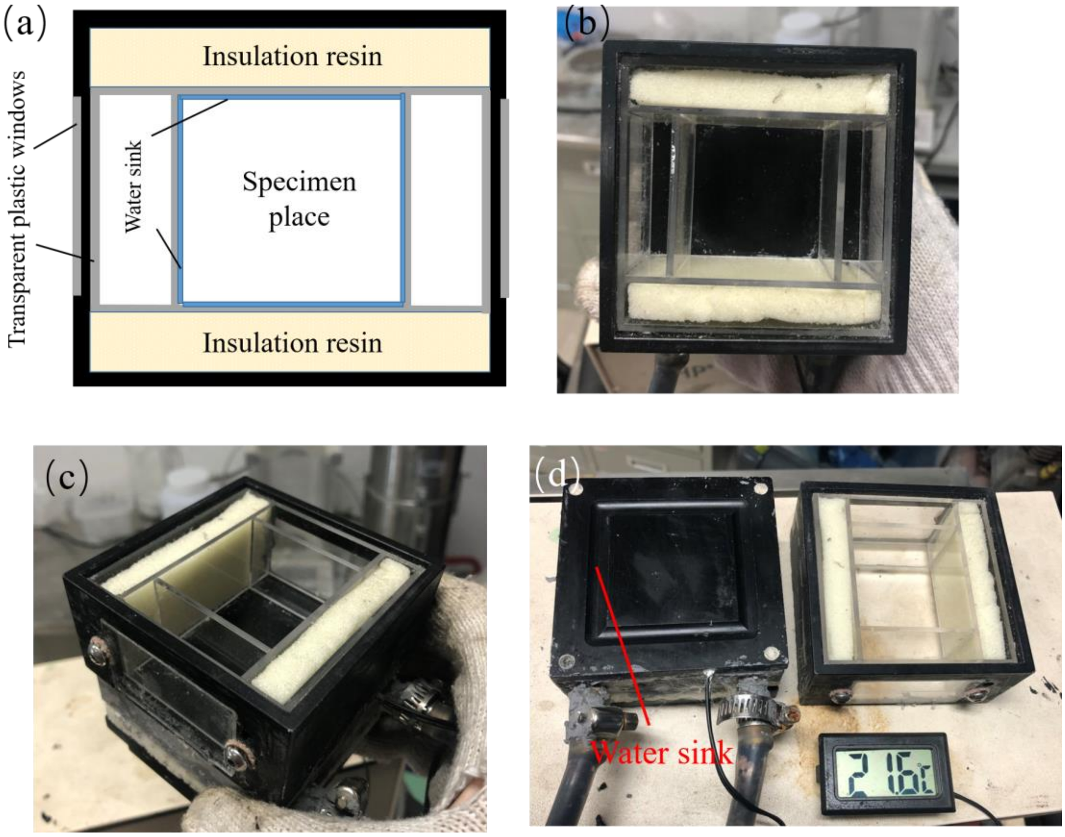

Figure S2. Structure details of the temperature-controlled measurement chamber: the 2D image (a) of the chamber structure; the vertical view (b) and side view (c) of the chamber structure; the water sink (d) around the specimen place.

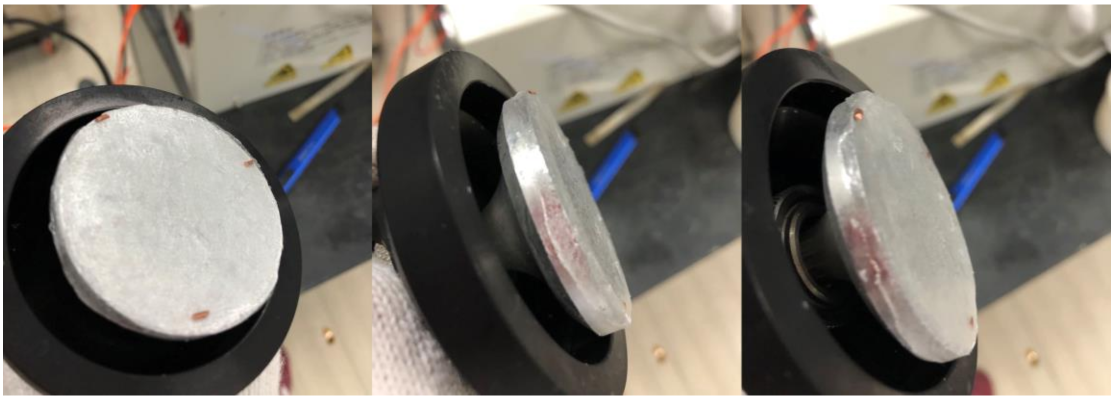

Figure S3. Ice layers adhered to the spindles after de-icing.

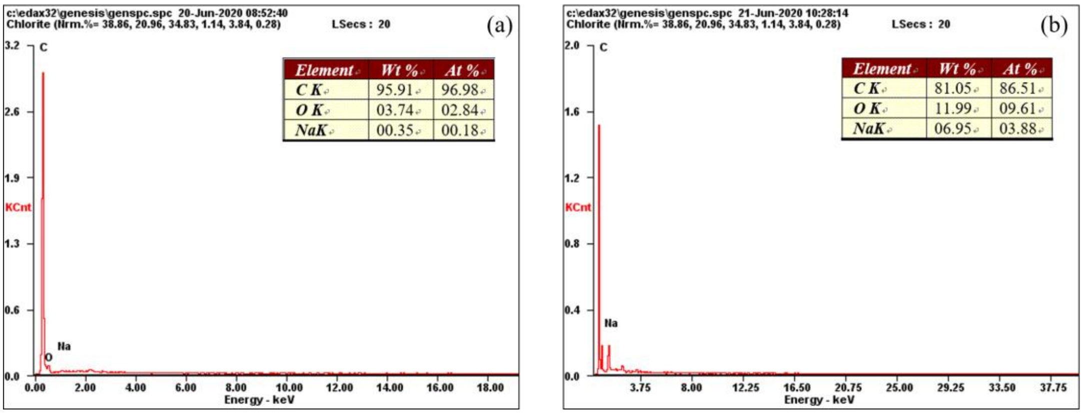

Figure S4. EDS spectra of the compound with 5% H-PAAS before (a) and after (b) water absorption.

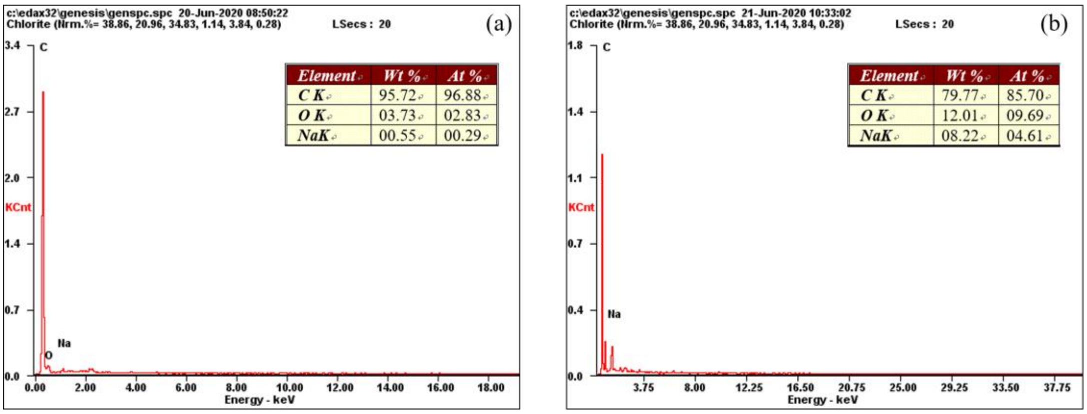

Figure S5. EDS spectra of the compound with 10% H-PAAS before (a) and after (b) water absorption.

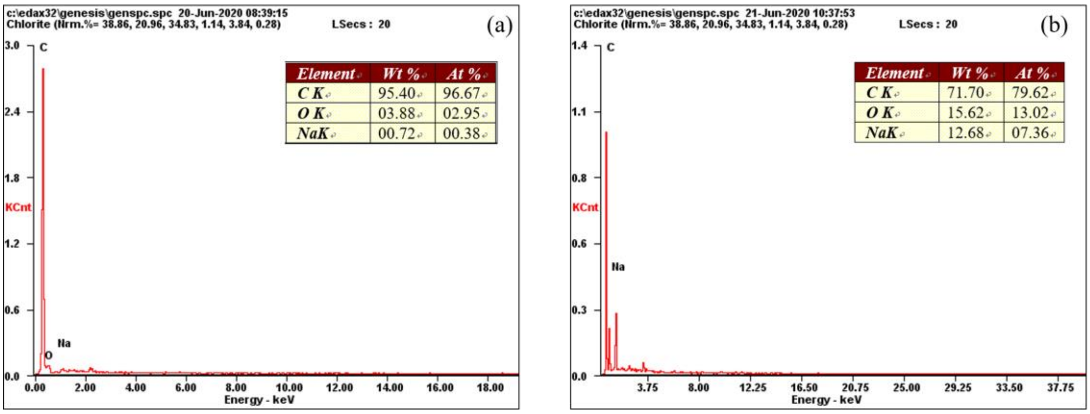

Figure S6. EDS spectra of the compound with 15% H-PAAS before (a) and after (b) water absorption.

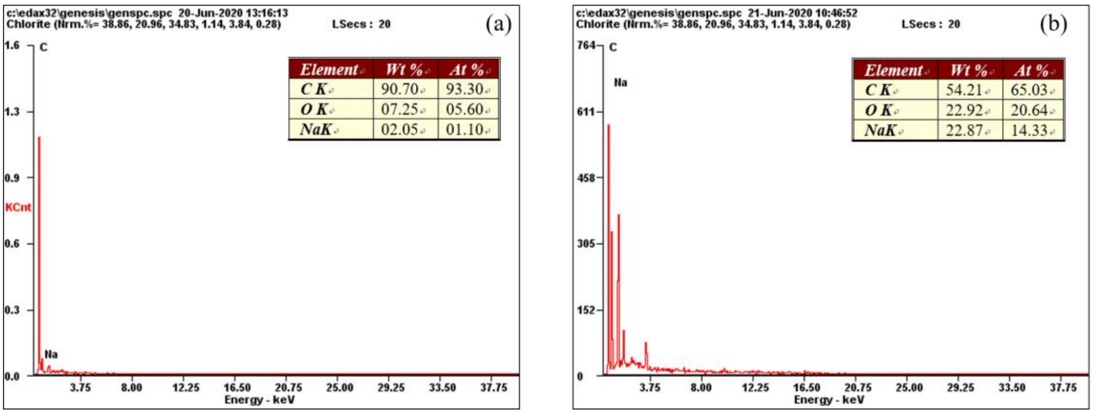

Figure S7. EDS spectra of the compound with 20% H-PAAS before (a) and after (b) water absorption.
